# Epidemiologic and Clinical Features of Mpox in Adults Aged >50 Years — United States, May 2022–May 2023

**DOI:** 10.15585/mmwr.mm7233a3

**Published:** 2023-08-18

**Authors:** Patrick C. Eustaquio, LaTweika A.T. Salmon-Trejo, Lisa C. McGuire, Sascha R. Ellington

**Affiliations:** ^1^CDC Mpox Emergency Response Team; ^2^Oak Ridge Institute for Science and Education, Oak Ridge, Tennessee; ^3^Division of Population Health, National Center for Chronic Disease Prevention and Health Promotion, CDC.

**Summary**

**What is already known about this topic?**

Childhood smallpox vaccination confers some cross-protection against mpox. Although persons aged >50 years likely received childhood smallpox vaccination, they might have more comorbidities and a higher risk for severe mpox compared with those aged ≤50 years. Information about waning cross-protective immunity and how this might affect risk for severe mpox is limited.

**What is added by this report?**

Among 29,984 adults with mpox, those aged >50 years had higher prevalences of immunocompromising conditions and HIV and lower prevalence of symptoms than did those aged ≤50 years. Among 1,020 adults aged >50 years with vaccination data, prevalences of pruritus, constitutional symptoms, and hospitalization were lower among those who received JYNNEOS vaccine than among those who had not.

**What are the implications for public health practice?**

All adults at risk for mpox should receive JYNNEOS vaccine, irrespective of childhood smallpox vaccination status.

## Abstract

During May 2022–May 2023, approximately 30,000 mpox cases were reported in the United States, predominantly among young adult men. Persons aged >50 years might experience more severe mpox disease because of a higher prevalence of comorbidities. Conversely, they could have residual protection from childhood smallpox vaccination against monkeypox virus infection and severe mpox, as has been suggested by investigation of some previous mpox outbreaks. To examine the characteristics of mpox cases among adults aged >50 years, analysts compared mpox epidemiology and clinical outcomes among all adults aged ≥18 years, by age group. Further, outcomes were compared among adults aged >50 years by JYNNEOS vaccination status. During May 10, 2022–May 17, 2023, among 29,984 adults with probable or confirmed mpox reported to CDC, 2,909 (9.7%) were aged >50 years, 96.3% of whom were cisgender men. Compared with adults aged 18–50 years, adults aged >50 years had higher prevalences of immunocompromising conditions (p<0.001) and HIV infection (p<0.001). Among adults with mpox aged >50 years, 27.6% had received JYNNEOS vaccination; this group had lower prevalences of constitutional symptoms (p<0.001), pruritus (p<0.001), and hospitalization (p = 0.002) compared with those who had not received JYNNEOS vaccine. Currently recommended JYNNEOS vaccination among all adults at risk for mpox should be encouraged, irrespective of childhood smallpox vaccination status.

## Introduction

During May 10, 2022–May 17, 2023, a total of 30,401 mpox cases* were reported in the United States, predominantly among young adult men. Adults aged >50 years likely received childhood smallpox vaccination, which was given routinely to children in the United States before being discontinued in 1971^†^; therefore, they might be less susceptible to monkeypox virus infection as a result of cross-protection (*1*). Childhood smallpox vaccination has been shown to provide partial protection against mpox, both in preventing monkeypox virus infection and severe mpox, in studies of earlier mpox outbreaks in the Democratic Republic of the Congo (*2*) and the United States (*3*). Because adults aged >50 years are likely to have more comorbidities than are younger adults, they might experience severe mpox disease, often with indications for treatment (*4*). This analysis described age group–specific epidemiologic characteristics and clinical mpox outcomes among adults and compared characteristics and outcomes among adults aged >50 years by recent JYNNEOS vaccination status.

## Methods

Data on confirmed or probable mpox cases^§^ among persons aged ≥18 years collected by jurisdictional public health departments and electronically reported through the National Notifiable Disease Surveillance System^¶^ or via a standardized case report form** during May 10, 2022–May 17, 2023, were included in the analysis. Adults with mpox were categorized into two age groups: 18–50 years and >50 years. Age-stratified categorical data, including epidemiologic characteristics, particularly sociodemographic characteristics, sexual contact during the 3 weeks preceding illness onset, HIV status, immunocompromising comorbidities excluding HIV (e.g., having undergone organ or stem cell transplant, active cancer, or immunosuppressive therapies), receipt of JYNNEOS vaccine, and clinical outcomes were summarized as frequencies and compared using Pearson’s chi-square or Fisher’s exact tests; p-values <0.05 were considered statistically significant. Adults who received ≥1 dose of JYNNEOS vaccine during the 2022 mpox outbreak were classified as vaccinated for the purposes of this report. To control for the potential effect of JYNNEOS vaccination on mpox clinical outcomes, analysts excluded adults with mpox who had received JYNNEOS when age-stratified clinical outcomes were being compared. An additional analysis among adults aged >50 years with mpox compared epidemiologic characteristics and clinical outcomes by JYNNEOS vaccination status; in this analysis, adults aged >50 years with mpox with unknown or missing JYNNEOS vaccination status or unknown or missing vaccination date were excluded. All statistical analyses were conducted using SAS software (version 9.4; SAS Institute). This analysis was reviewed by CDC and was conducted consistent with federal law and applicable CDC policy.^††^

## Results

Among 29,984 adults with mpox reported by May 17, 2023, 2,909 (9.7%) were aged >50 years, including 2,794 (96.3%) who were cisgender men (Table 1). Among those aged >50 years, 1,297 (47.3%) were non-Hispanic White (White), 561 (20.5%) were non-Hispanic Black or African American (Black), and 758 (27.7%) were Hispanic or Latino (Hispanic). Among the 27,075 adults with mpox aged 18–50 years, 7,085 (27.8%) were White, 8,733 (34.2%) were Black, and 7,878 (30.9%) were Hispanic (p<0.001). Reports of any sexual contact during the 3 weeks preceding illness onset was similar among adults with mpox aged 18–50 years (78.6%) and those aged >50 years (77.0%). Among cisgender men with mpox, who accounted for 95.2% and 96.3% of adults with mpox aged 18–50 years and >50 years, respectively, 96.6% of these contacts among those aged 18–50 years, and 97.0% among those aged >50 years, were with other cisgender men. Immunocompromising conditions were more prevalent overall among adults aged >50 years (15.0%) than among those aged 18–50 years (11.1%), as was receipt of JYNNEOS vaccine (27.6% and 21.3%, respectively). Examination of symptoms and outcomes among adults with mpox who had not received JYNNEOS vaccine found lower prevalences of gastrointestinal (rectal or abdominal)^§§^ and constitutional symptoms^¶¶^ among those aged >50 years (37.3% and 85.0%, respectively) than among those aged 18–50 years (50.3% and 91.2%, respectively); the prevalences of hospitalization and death were comparable between the two age groups.

**TABLE 1:** Demographic and epidemiologic characteristics of adults with mpox, by age group (N = 29,984) — United States, May 10, 2022–May 17, 2023

Characteristic and clinical outcome	Age group, no. (column %)*		p-value^†^
	18–50 yrs n = 27,075	>50 yrs n = 2,909	
**Gender identity**			
Cisgender men	25,681 (95.2)	2,794 (96.3)	<0.001
Cisgender women	809 (3.0)	88 (3.0)	
Transgender men	55 (0.2)	0 (—)	
Transgender women	220 (0.8)	9 (0.3)	
Other gender identity	225 (0.8)	11 (0.4)	
Missing	85	7	
**Race and ethnicity^§^**			
Black or African American	8,733 (34.2)	561 (20.5)	<0.001
White	7,085 (27.8)	1,297 (47.3)	
Hispanic or Latino	7,878 (30.9)	758 (27.7)	
Other	1,684 (6.6)	120 (4.4)	
Multiracial	134 (0.5)	4 (0.1)	
Missing	1,561	169	
**Any sexual contact during the 3 wks preceding illness onset**			
Yes	15,495 (78.6)	1,618 (77.0)	0.083
No	4,216 (21.4)	484 (23.0)	
Missing	7,364	807	
**Sexual partner among cisgender men^¶^**			
Cisgender men only	12,561 (93.5)	1,393 (94.8)	0.201
Cisgender men and other genders	419 (3.1)	32 (2.2)	
Exclude cisgender men	454 (3.4)	45 (3.1)	
Missing	12,247	1,324	
**HIV status**			
HIV positive	4,798 (55.4)	552 (66.2)	<0.001
HIV negative	3,860 (44.6)	282 (33.8)	
Unknown HIV status or missing	18,417	2,075	
Immunocompromising condition**			
Yes	1,308 (11.1)	185 (15.0)	<0.001
No	10,466 (88.9)	1,045 (85.0)	
Missing	15,301	1,679	
**Received ≥1 dose of JYNNEOS vaccine**			
Yes	2,481 (21.3)	282 (27.6)	<0.001
No	9,177 (78.7)	738 (72.4)	
Missing	15,417	1,889	
**Rash^††^**			
Yes	6,867 (96.8)	563 (96.6)	0.72
No	224 (3.2)	20 (3.4)	
Missing	2,086	155	
**Pruritus^††^**			
Yes	2,972 (58.7)	221 (59.4)	0.78
No	2,092 (41.3)	151 (40.6)	
Missing	4,113	366	
**Rectal symptoms^††,§§^ or abdominal pain**			
Yes	2,719 (50.3)	134 (37.3)	<0.001
No	2,683 (49.7)	225 (62.7)	
Missing	3,775	379	
**Constitutional symptoms^††,¶¶^**			
Yes	6,103 (91.2)	424 (85.0)	<0.001
No	586 (8.8)	75 (15.0)	
Missing	2,488	239	
**Hospitalization^††^**			
Yes	657 (7.5)	58 (8.3)	0.459
No	8,110 (92.5)	644 (91.7)	
Missing	410	36	
**Death^††^**			
Yes	12 (0.2)	3 (0.6)	0.069
No	6,488 (99.8)	532 (99.4)	
Missing	2,677	203	

* Percentages were calculated using nonmissing data.

^†^ Pearson’s chi-square or Fisher’s exact test.

^§^ Persons of Hispanic or Latino (Hispanic) origin might be of any race but are categorized as Hispanic; all racial groups are non-Hispanic.

^¶^ Only among cisgender men aged 18–40 years (25,681) and >50 years (2,794).

** Excluding HIV infection and AIDS.

^††^ Clinical outcomes were compared by age group after excluding adults who received JYNNEOS vaccine (2,763).

^§§^ Includes rectal pain/bleeding, pus in the stool, proctitis, or tenesmus.

^¶¶^ Includes fever, headache, malaise, myalgia, chills, or lymphadenopathy.

Among 1,020 adults with mpox aged >50 years with known JYNNEOS vaccination status, the prevalences of HIV and immunocompromising conditions were similar among those who did and did not receive JYNNEOS vaccine (Table 2). Among those aged >50 years, the prevalences of constitutional symptoms (64.9%), pruritus (44.1%), and hospitalization (1.2%) among those who had received JYNNEOS vaccine were lower than were those among persons who did not receive vaccine (81.3%, 56.9%, and 7.4%, respectively).

**TABLE 2:** Characteristics, symptoms, and clinical outcomes among adults aged >50 years with mpox and known JYNNEOS vaccination status,* by vaccination status (N = 1,020) — United States, May 10, 2022–May 17, 2023

Characteristic or outcome	Received JYNNEOS vaccine, no. (column %)^†^		p-value^§^
	No n = 738	Yes n = 282	
**HIV status**			
Positive	492 (66.8)	60 (61.9)	0.974
Negative	245 (33.2)	37 (38.1)	
Unknown or missing	1,994	81	
**Immunocompromising condition^¶^**			
Yes	172 (15.0)	13 (14.9)	0.356
No	971 (85.0)	74 (85.1)	
Missing	1,588	91	
Constitutional symptoms**			
Yes	1,320 (81.3)	74 (64.9)	<0.001
No	303 (18.7)	40 (35.1)	
Missing	1,108	64	
**Rash**			
Yes	1,928 (97.7)	123 (93.9)	0.895
No	45 (2.3)	8 (6.1)	
Missing	758	47	
**Pruritus**			
Yes	489 (56.9)	41 (44.1)	<0.001
No	371 (43.1)	52 (55.9)	
Missing	1,871	85	
**Rectal symptoms^††^ or abdominal pain**			
Yes	451 (36.3)	43 (43.0)	0.486
No	791 (63.7)	57 (57.0)	
Missing	1,489	78	
**Hospitalization**			
Yes	163 (7.4)	2 (1.2)	0.002
No	2,044 (92.6)	160 (98.8)	
Missing	524	16	
**Death**			
Yes	5 (0.3)	0 (—)	0.446
No	1,647 (99.7)	100 (100.0)	
Missing	1079	78	

* A total of 1,889 (65%) mpox patients were excluded because vaccination status (1662; 57%) or date (227; 8%) was missing or unknown.

^†^ Percentages were calculated using nonmissing data.

^§^ Pearson’s chi-square or Fisher’s exact test.

^¶^ Excluding HIV infection and AIDS.

** Includes fever, headache, malaise, myalgia, chills, or lymphadenopathy.

^††^ Includes rectal pain/bleeding, pus in the stool, proctitis, or tenesmus.

## Discussion

Mpox might affect multiple organ systems; therefore, persons with comorbidities, including HIV and immunocompromising conditions, which are more prevalent among adults aged >50 years, might be at increased risk for more severe mpox disease (*4*). Most deaths and hospitalizations among adults with mpox in the United States during the recent outbreak occurred in persons with immunocompromising conditions (*5*,*6*). In this report, however, excluding adults who had received JYNNEOS vaccine, the prevalences of hospitalization and death among adults aged >50 years with mpox were similar to those in persons aged 18–50 years, and the prevalences of some symptoms were lower among adults aged >50 years than among those aged 18–50 years. Although the reasons for this finding are not clear, receipt of smallpox vaccine as part of childhood immunization before routine smallpox vaccination discontinued in the United States in 1971 might confer some level of protection against mpox in the current outbreak, with respect to whether the clinical presentation was atypical or asymptomatic (*7*).

The prevalences of pruritus, constitutional symptoms, and hospitalizations were lower among adults with mpox aged >50 years who received JYNNEOS vaccine than among those who did not receive the vaccine. Although childhood smallpox vaccination might confer some protection against monkeypox virus infection and might mitigate mpox disease severity, it is likely that receiving the currently recommended JYNNEOS vaccine provided additional protection. This finding is consistent with a study among adults who received JYNNEOS vaccine, wherein adults who received ≥1 dose of JYNNEOS vaccine had lower rates of hospitalization and symptoms (*8*). This finding underscores the importance that persons at risk for mpox, particularly adults aged >50 years, receive currently recommended JYNNEOS vaccination. Immunologic studies have demonstrated some long-term immunologic memory from childhood smallpox vaccination that is cross-protective against mpox (*7*,*9*), but such immunity might have waned (*7*). JYNNEOS vaccination is recommended for all adults who are at risk for mpox irrespective of receipt of childhood smallpox vaccination (*10*).

### Limitations

The findings in this report are subject to at least four limitations. First, data for some variables, such as HIV status, presence of immunocompromising conditions and mpox symptoms, and JYNNEOS vaccination status were frequently missing in national case surveillance data, which limits full characterization of the epidemiologic and clinical features among adults with mpox and could contribute to confounding bias. Second, some variables were self-reported, and might be subject to recall and social desirability biases. Third, receipt of smallpox vaccination was assumed for all adults aged >50 years despite nuances in implementation in the United States and other countries of origin of adults with mpox. Finally, this analysis was limited to confirmed and probable mpox cases reported to jurisdictional public health departments and might not represent all adults with mpox.

### Implications for Public Health Practice

Hospitalization was less likely among adults aged >50 years with mpox who had received JYNNEOS vaccine than among those who had not. All adults who are at risk for acquiring mpox, regardless of childhood smallpox vaccination status, should receive 2 doses of JYNNEOS vaccine (*10*).

## Footnotes

* Includes cases from all age groups. https://www.cdc.gov/poxvirus/mpox/response/2022/index.html (Accessed May 17, 2023).

† Routine childhood smallpox vaccination ended in 1971 in the United States, although vaccination continued for some persons because of implementation nuances and recommendations based on risk. https://www.cdc.gov/mmwr/preview/mmwrhtml/rr6402a1.htm

§ Cases include positive test results for either *Monkeypox virus* or *Orthopoxvirus*.

¶
https://www.cdc.gov/nndss/index.html

**
https://www.cdc.gov/poxvirus/mpox/pdf/sCRF-Short-Form.pdf

†† 45 C.F.R. part 46.102(l)(2), 21 C.F.R. part 56; 42 U.S.C. Sect. 241(d); 5 U.S.C. Sect. 552a; 44 U.S.C. Sect. 3501 et seq.

§§ Includes rectal pain/bleeding, pus in the stool, proctitis, or tenesmus.

¶¶ Includes fever, headache, malaise, myalgia, chills, or lymphadenopathy.

## References

[1] Jacobs BL, Langland JO, Kibler KV, Vaccinia virus vaccines: past, present and future. Antiviral Res 2009;84:1–13. 10.1016/j.antiviral.2009.06.006 19563829 PMC2742674

[2] Rimoin AW, Mulembakani PM, Johnston SC, Major increase in human monkeypox incidence 30 years after smallpox vaccination campaigns cease in the Democratic Republic of Congo. Proc Natl Acad Sci U S A 2010;107:16262–7. 10.1073/pnas.1005769107 20805472 PMC2941342

[3] Karem KL, Reynolds M, Hughes C, Monkeypox-induced immunity and failure of childhood smallpox vaccination to provide complete protection. Clin Vaccine Immunol 2007;14:1318–27. 10.1128/CVI.00148-07 17715329 PMC2168110

[4] Reynolds MG, McCollum AM, Nguete B, Shongo Lushima R, Petersen BW. Improving the care and treatment of monkeypox patients in low-resource settings: applying evidence from contemporary biomedical and smallpox biodefense research. Viruses 2017;9:380. 10.3390/v9120380 29231870 PMC5744154

[5] Miller MJ, Cash-Goldwasser S, Marx GE, ; CDC Severe Monkeypox Investigations Team. Severe monkeypox in hospitalized patients—United States, August 10–October 10, 2022. MMWR Morb Mortal Wkly Rep 2022;71:1412–7. 10.15585/mmwr.mm7144e1 36327164 PMC9639440

[6] Riser AP, Hanley A, Cima M, Epidemiologic and clinical features of mpox-associated deaths—United States, May 10, 2022–March 7, 2023. MMWR Morb Mortal Wkly Rep 2023;72:404–10. 10.15585/mmwr.mm7215a5 37053126 PMC10121256

[7] Sanz-Muñoz I, Sánchez-dePrada L, Sánchez-Martínez J, Possible mpox protection from smallpox vaccine-generated antibodies among older adults. Emerg Infect Dis 2023;29:656–8. 10.3201/eid2903.221231 36732061 PMC9973709

[8] Farrar JL, Lewis NM, Houck K, ; Mpox Cases in Vaccinated Persons Team. Demographic and clinical characteristics of mpox in persons who had previously received 1 dose of JYNNEOS vaccine and in unvaccinated persons—29 US Jurisdictions, May 22–September 3, 2022. MMWR Morb Mortal Wkly Rep 2022;71:1610–5. 10.15585/mmwr.mm715152a2 36580416 PMC9812445

[9] Adamo S, Gao Y, Sekine T, Memory profiles distinguish cross-reactive and virus-specific T cell immunity to mpox. Cell Host Microbe 2023;31:928–936.e4. 10.1016/j.chom.2023.04.015 37236191 PMC10211501

[10] CDC. Mpox: mpox vaccination basics. Atlanta, GA: US Department of Health and Human Services; CDC; 2022. Accessed August 14, 2023. https://www.cdc.gov/poxvirus/mpox/vaccines/vaccine-basics.html

